# Hypertension unawareness among Chinese patients with first-ever stroke

**DOI:** 10.1186/s12889-016-2835-1

**Published:** 2016-02-19

**Authors:** Qinqin Cao, Pei Pei, Jun Zhang, Jillian Naylor, Xinying Fan, Biyang Cai, Qiliang Dai, Wen Sun, Ruidong Ye, Ruifeng Shi, Keting Liu, Yongjun Jiang, Wenhua Liu, Fang Yang, Wusheng Zhu, Yunyun Xiong, Xinfeng Liu, Gelin Xu

**Affiliations:** Department of Neurology, Jinling Hospital, Medical School of Nanjing University, 305 East Zhongshan Road, Nanjing, 210002 Jiangsu Province China; School of Humanity, Ningbo Dahongying University, Ningbo, 315175 Zhejiang Province China; Department of Neurology, Melbourne Brain Centre, Royal Melbourne Hospital, University of Melbourne, Melbourne, Victoria Australia; Department of Neurology, Jinling Hospital, Southern Medical University, Nanjing, 210002 Jiangsu Province China

**Keywords:** China, Hypertension unawareness, Patient education, Risk factors, Stroke

## Abstract

**Background:**

The low rates of hypertension treatment and control, partly due to its unawareness, are the main causes of the high stroke incidence in China. The purpose of this study was to evaluate hypertension unawareness amongst patients with first-ever stroke and to detect factors associated with its unawareness.

**Methods:**

We selected those diagnosed with hypertension from patients with first-ever stroke registered in the Nanjing Stroke Registry Program between 2004 and 2014. These hypertensives were divided as being aware or unaware of their hypertension by using a brief questionnaire conducted shortly after the stroke. Multivariate logistic regression analysis was performed to identify potential factors associated with hypertension unawareness.

**Results:**

Of the 5309 patients with first-ever stroke, 3732 (70.3 %) were diagnosed with hypertension. Among which, 593 (15.9 %) were unaware of their hypertension at the time of stroke onset. Lower-level of education (primary school or illiteracy) and smoking were associated positively with hypertension unawareness; while advanced age, overweight, diabetes mellitus, heart diseases and family history of stroke were associated negatively with hypertension unawareness. Annual data analyzed indicated that the rate of hypertension awareness increased during the past 11 years (*r* = 0.613, *P* = 0.045 for trends).

**Conclusions:**

A substantial proportion (15.9 %) of Chinese patients with hypertension had not been aware of this covert risk until an overt stroke occurred. Hypertension unawareness was associated with lower educational levels and smoking, which address the importance of health education especially in these individuals.

## Background

Hypertension is the leading modifiable risk factor for cardiovascular disease, involved in approximately 54 % of stroke cases and 47 % of ischemic heart disease incidences worldwide [1]. With 10-mm Hg lowering in systolic blood pressure (SBP), the relative risk for major cardiovascular events can be reduced by 20 to 25 %, with more remarkable effects on stroke than coronary outcomes [2]. Unfortunately, the control rate of hypertension is as low as 5 % in China [3]. This prevalence of uncontrolled hypertension has been associated with the high stroke incidence and mortality in developing countries [2, 4]. Unawareness of elevated blood pressure may be a major contributing factor for the inappropriate control of hypertension, absence of anti-hypertension treatments (including behavioral interventions) and improper monitoring of blood pressure even with treatments. Exploring possible factors associated with unawareness of elevated blood pressure is of vital importance, therefore, for preventing cardiovascular diseases and other hypertension related conditions in the population.

Most of the previous studies on hypertension awareness, treatment and control were conducted in communities or in general populations. Few studies have investigated the hypertension unawareness in stroke patients, especially patients with first-ever stroke. Considering the high prevalence of untreated and uncontrolled hypertension in China [3, 5, 6], it is possible that a substantial proportion of hypertensives have been unaware of their elevated blood pressure until a stroke occurred. It is reasonable to predict that stroke incidence may be reduced if hypertension awareness is strengthened. To evaluate the magnitude of unawareness and elucidate possible correlates, we assessed hypertension unawareness in a large Chinese patient group with first-ever stroke.

## Methods

### Study subjects

Subjects in this study were patients with first-ever stroke enrolled in the Nanjing Stroke Registry Program (NSRP) between January 2004 and December 2014. The NSRP, established in July 2002, is the first hospital-based stroke registry program in the Mainland. Its central base is in Jinling Hospital, Nanjing, a city located in the southeast area of China. Detailed procedures for patient enrollment in NSRP have been published [7]. Patients were eligible for this study if they had a first-ever stroke confirmed by a CT or MRI scan within 14 days of onset, aged 18 years or above, and survived 2 weeks after the index stroke. Written informed consent was obtained from each patient and the study was approved by the Ethics Committee in Jinling Hospital.

### Definitions

Stroke was defined by the World Health Organization (WHO) as rapidly developed clinical signs of focal (or global) disturbance of cerebral function, lasting for more than 24 h or being interrupted by death within 24 h, with no apparent cause other than of vascular origin [8]. Hypertension was defined as an average SBP ≥140 mm Hg or an average diastolic blood pressure (DBP) ≥90 mm Hg on at least three measurements after the first week of stroke onset, or receiving pharmacological treatment for hypertension [9].

### Blood pressure monitoring

To determine the status of blood pressure at time of stroke onset, all patients were scheduled for blood pressure monitoring at least twice daily within 14 days of stroke onset. For those discharged from hospital earlier than the 15th day of stroke onset, blood pressure monitoring continued in local clinics, nursing homes, or at homes by a trained family member with an electronic sphygmomanometer (preferably Omron HEM7051). The results were collected during a subsequent clinical visit. Management of blood pressure in patients with acute stroke was in accordance with guidelines. For example, the blood pressure should not be lowered in the acute stage of stroke unless it is higher than 220/120 mmHg [10].

### Evaluation of blood pressure awareness

To evaluate the awareness of blood pressure status, each patient enrolled was assessed with a brief questionnaire shortly after stroke admission. Issues in the questionnaire included experiences and frequency of blood pressure measurement before the index stroke, numeric SBP and DBP if measured, awareness of a hypertension diagnosis raised by a physician, any antihypertensive medication usage within 2 weeks before the index stroke, blood pressure responsiveness to the treatments and history of cardiovascular disease other than stroke. The questionnaire was preferably responded by the patient. In case of coma or advanced cognitive impairment, a relative or guardian who was familiar with the medical history of the patient was encouraged to participate in the questionnaire survey. To exclude possible memory bias, the survey was conducted before blood pressure monitoring.

### Stratification of blood pressure status

For all patients, results of blood pressure monitoring and history of antihypertensive medication were considered for the diagnosis of hypertension at time of stroke onset. Patients diagnosed with hypertension were grouped as being aware or unaware of this condition based on their responses to the questionnaire. Those who were aware of the hypertension were stratified as treated or untreated, and those being treated were further stratified as controlled or uncontrolled. Being aware of hypertension indicated that the patient or family members recalled a hypertension diagnosis made by a physician. Being treated for hypertension indicated that the patient took at least one antihypertensive medication for elevated blood pressure within 2 weeks immediately before the index stroke [3, 6, 11]. When the DBP <90 mm Hg and the SBP <140 mm Hg, hypertension was regarded as being controlled [12].

### Possible influencing factors

Data of demographic profiles (age, sex, marriage status), major risk factors for cardiovascular disease other than hypertension (diabetes mellitus (DM), hyperlipidaemia, smoking, alcohol drinking, overweight), type of index stroke, history of heart diseases, socioeconomic status (resident environment, occupation, educational level, possession of health insurance) and family history of stroke were collected from NSRP and analyzed as potential influencing factors associated with hypertension unawareness. Smoking was defined as that patient had smoked more than 1 cigarette per day for at least 6 months. Alcohol drinking was defined as that patient consumed more than two drinks a day for at least 2 months. Overweight was defined as a body mass index (BMI) ≥25 kg/m^2^. Presences of DM, heart diseases and hyperlipidaemia were confirmed by medical records.

### Statistical analysis

Kolmogorov-Smirnov test was performed to determine whether the distribution of a continuous parameter was normal. Continuous variables were presented as median (interquartile range [IQR]), whilst categorical variables were presented as frequencies. For inter-group comparisons, continuous variables were analyzed with Mann–Whitney *U* test, whilst categorical variables were analyzed with *χ*^*2*^ test or Fisher’s exact test. To identify possible factors associated with hypertension unawareness, the significant variables (*P* < 0.1) identified in univariate analyses were entered in a multivariate logistic regression model for further analysis. Results were presented as odds ratio (OR) and 95 % confidence interval (CI). The linear regression analysis was performed to test the temporal trends of hypertension awareness, treatment and control over years. *P* < 0.05 for two-sided test was deemed as statistical significance. All analyses were conducted using SPSS software version 17.0 (SPSS Inc, Chicago, IL).

## Results

The study screened 5309 patients with first-ever stroke registered in NSRP between 2004 and 2014. Based on the results of blood pressure monitoring and history of antihypertensive medication usage, 3732 (70.3 %) patients were confirmed with hypertension at time of stroke onset. These 3732 patients were selected as subjects for the analyses of hypertension unawareness and the factors associated.

Of all 3732 hypertensive patients chosen, 3367 (90.2 %) were diagnosed with ischemic stroke, 222 (6.0 %) with intracerebral hemorrhage and 143 (3.8 %) with subarachnoid hemorrhage. At time of stroke onset, 3139 (84.1 %) patients were aware of their hypertension through a previous diagnosis, whilst 593 (15.9 %) were unaware; 1534 (41.1 %) received anti-hypertension treatment, and 658 (17.6 %) had their blood pressure controlled.

After dichotomizing patients as being aware or unaware of hypertension at time of stroke onset, data of demographic profiles, major risk factors for stroke, socioeconomic status and family history of stroke from each group were compared using univariate analysis (Table 1). In contrast with patients who were aware of their hypertension, those who were unaware were younger (median 60 vs 62 years, *P* = 1.31 × 10^−4^) and had a lower BMI (median 24.0 vs 24.5, *P* = 3.47 × 10^−5^). They also had a lower prevalence of DM (13.7 % vs 27.8 %, *P* = 4.34 × 10^−14^), heart diseases (5.4 % vs 11.0 %, *P* = 1.44 × 10^−5^) and family history of stroke (4.6 % vs 11.1 %, *P* = 1.55 × 10^−7^). Furthermore, patients who were smokers (42.0 % vs 35.5 %, *P* = 0.003), engaged in manual work (62.6 % vs 53.2 %, *P* = 2.78 × 10^−5^), lack of health insurance (55.0 % vs 49.8 %, *P* = 0.022), with lower-level of education (primary school or illiteracy, 38.0 % vs 27.3 %, *P* = 3.35 × 10^−7^), and lived in rural areas (38.4 % vs 27.9 %, *P* = 5.23 × 10^−7^) were less likely to be aware of their hypertension.

**Table 1:** Baseline characteristics of stroke patients by hypertension awareness

	Aware (*n* = 3139)	Unaware (*n* = 593)	*P* value
Age, median (IQR)	62 (54–70)	60 (50–69)	1.31 × 10^−4^
Age ≥ 60 years, n (%)	1835 (58.5)	300 (50.6)	4.14 × 10^−4^
Male, n (%)	2186 (69.6)	412 (69.5)	0.961
Married, n (%)	3135 (99.9)	592 (99.8)	0.579
BMI, median (IQR)	24.5 (22.9–26.0)	24.0 (22.5–25.4)	3.47 × 10^−5^
BMI ≥ 25, n (%)	1242 (39.6)	198 (33.4)	0.005
Smoking, n (%)	1115 (35.5)	249 (42.0)	0.003
Alcohol drinking, n (%)	682 (21.7)	142 (23.9)	0.235
Diabetes mellitus, n (%)	874 (27.8)	81 (13.7)	4.34 × 10^−14^
Hyperlipidaemia, n (%)	469 (14.9)	76 (12.8)	0.205
Heart diseases, n (%)	345 (11.0)	32 (5.4)	1.44 × 10^−5^
History of TIA, n (%)	123 (3.9)	18 (3.0)	0.348
Lack of health insurance, n (%)	1564 (49.8)	326 (55.0)	0.022
Rural residency, n (%)	877 (27.9)	228 (38.4)	5.23 × 10^−7^
Lower-level of education, n (%)	806 (27.3)	222 (38.0)	3.35 × 10^−7^
Manual work, n (%)	1663 (53.2)	371 (62.6)	2.78 × 10^−5^
Family history of stroke, n (%)	349 (11.1)	27 (4.6)	1.55 × 10^−7^

*IQR* indicates interquartile ranges, *BMI* indicates body mass index, *TIA* indicates transient ischemic attack. Lower-level of education indicates primary school or illiteracy

To determine potential factors attributable to hypertension unawareness, those parameters manifested significant difference (*P* < 0.1) between groups were further analyzed with multivariate logistic regression (Table 2). Smokers (OR: 1.38; 95 % CI: 1.11 to 1.71) and those with lower-level of education (OR: 1.61; 95 % CI: 1.27 to 2.03) were less likely to be aware of their hypertension. Whilst patients with advanced age (OR: 0.69; 95 % CI: 0.56 to 0.83), overweight (OR: 0.77; 95 % CI: 0.64 to 0.94), DM (OR: 0.44, 95 % CI: 0.34 to 0.57), heart diseases (OR: 0.54, 95 % CI: 0.37 to 0.80) or with family history of stroke (OR: 0.38, 95 % CI: 0.25 to 0.57) were more likely to be aware of their hypertension.

**Table 2:** Factors associated with hypertension unawareness

	Hypertension unawareness	
Variables	OR (95 % CI)	*P* value
Age ≥60y	0.69 (0.56 to 0.83)	0.0002
Male	0.93 (0.73 to 1.17)	0.518
BMI ≥ 25	0.77 (0.64 to 0.94)	0.009
Smoking	1.38 (1.11 to 1.71)	0.004
Heart diseases	0.54 (0.37 to 0.80)	0.002
Diabetes mellitus	0.44 (0.34 to 0.57)	2.53 × 10^−10^
Lack of health insurance	0.93 (0.75 to 1.15)	0.489
Rural residency	1.23 (0.96 to 1.57)	0.103
Lower-level of education	1.61 (1.27 to 2.03)	0.73 × 10^−4^
Manual work	1.13 (0.90 to 1.41)	0.282
Family history of stroke	0.38 (0.25 to 0.57)	2.58 × 10^−6^

Parameters were analyzed with multivariate logistic regression analysis. *OR* indicates odds ratio, *CI* indicates confidence interval, *BMI* indicates body mass index

**Fig. 1:**
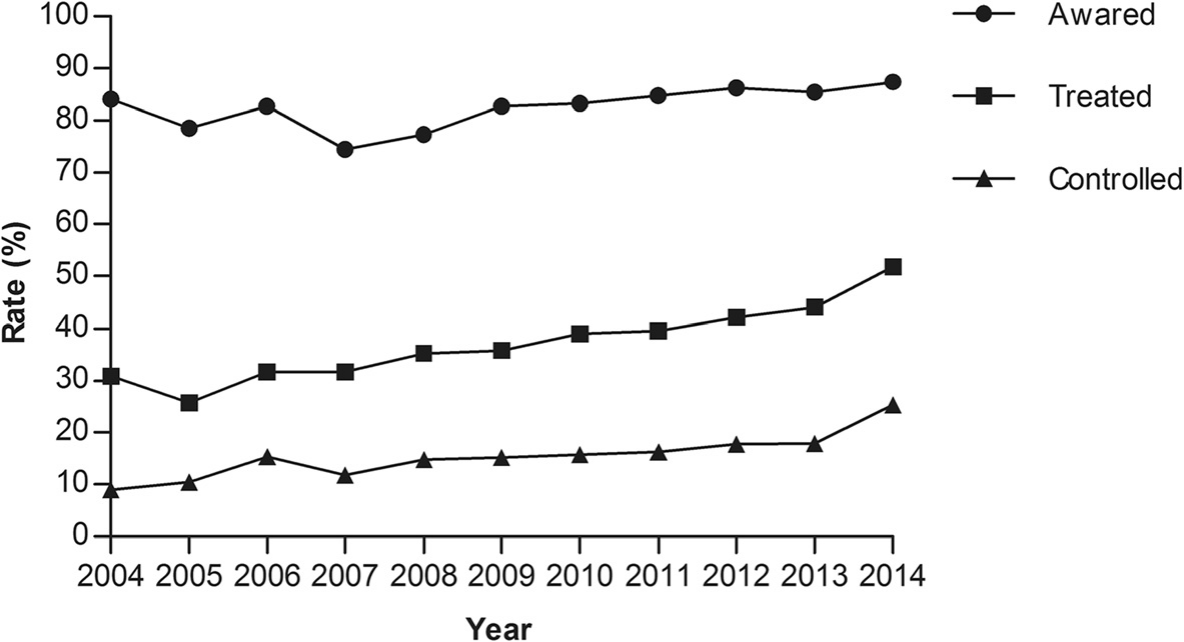
Trends in awareness, treatment, and control of hypertension in stroke patients, 2004–2014. There had been significant increases in hypertension awareness (*r* = 0.613, *P* = 0.045 for trends), treatment (*r* = 0.947, *P* = 9.89 × 10^−6^ for trends), and control (*r* = 0.890, *P* = 2.39 × 10^−4^ for trends) in this stroke patient cohort during the 11 years

To assess the temporal trends of hypertension awareness, treatment, and control, we evaluated these rates over the years (Fig. 1). Linear regression indicated the rate of awareness increased slightly but significantly in 11 years (from 84.0 % in 2004 to 87.4 % in 2014, *r* = 0.613, *P* = 0.045 for trends). Although rates of hypertension treatment (from 30.8 % in 2004 to 51.9 % in 2014, *r* = 0.947, *P* = 9.89 × 10^−6^ for trends) and control (from 9.0 % in 2004 to 25.3 % in 2014, *r* = 0.890, *P* = 2.39 × 10^−4^ for trends) at time of stroke onset increased, there was considerable margin for improvement due to the extensive hypertension unawareness.

## Discussion

This study presents several important findings regarding hypertension unawareness, associated factors as well as hypertension treatment and control in China. A substantial proportion (15.9 %) of Chinese patients with hypertension had not been aware of this covert risk until an overt stroke occurred. Lower-level of education and smoking were associated with hypertension unawareness, whilst advanced age, overweight, DM, heart diseases and family history of stroke were associated with improved hypertension awareness.

As expected, this study associated lower-level of education (primary school or illiteracy) with hypertension unawareness in Chinese stroke patients. Patients with lower-level of education may lack sufficient drive and/or adequate skills to monitor their blood pressure over an extended period of time [13–15]. Their knowledge of hypertension as a risk factor for cardiovascular disease may be limited compared with that of those with advanced education [16–18]. They are more prone to disregard health education programs as available [13]. Poorly educated patients, therefore, may be less likely to have their blood pressure measured regularly [16]. Conversely, the promoting effects of advanced education on hypertension awareness have been observed in other studies [11, 19].

In this study, hypertension unawareness was more prevalent in young than in elderly patients. Young people may be less vigilant to the hazardous effects of elevated blood pressure to their health. Many other studies also reported that young people have a lower rate of hypertension awareness [11, 19–21].

Compared with non-smoking patients, smokers were less likely to be aware of their hypertension. This finding was in consistent with results from some community-based studies [20, 22], but contrary to those from others [11, 19]. This finding highlighted the importance of hypertension education in smoking individuals, which may be more feasible and effective in communities. Still other studies have reported that women had a higher level of hypertension awareness than men, however in this study we did not detect different hypertension awareness between sexes [11, 19, 20]. Selection bias may be responsible for these discrepancies, for example, stroke patients enrolled in this study showed a male predominance (69.6 %), while the sex distribution of community populations were more even. No significant correlation between health insurance and hypertension unawareness was detected in this study. The socioeconomic development was remarkable in recent years, but the situation that patients are responsible for their own health remained largely unchanged in Nanjing and also in China. Although more citizens especially those in urban were covered by the government-run health insurance in recent 10 years, there was no regular blood pressure-checking plan even for patients with hypertension. Patients, with or without health insurance, usually had their blood pressure being checked at their own will.

In this study, patients with comorbidities, such as DM, heart diseases and overweight were more likely to be aware of their hypertension. These concomitant chronic conditions usually require regular follow-ups, thus the occasions of patients meeting with their healthcare providers may increase, so does the chance of a covert hypertension being diagnosed. Patient’s experiences in diagnosing and managing DM, heart diseases and overweight may help for improving blood pressure administration. Other chronic diseases may also alert the patients with the possible catastrophic outcomes of uncontrolled hypertension, therefore reinforcing their drive for blood pressure monitoring and management. These synergetic effects of concomitant chronic diseases on hypertension management were also reported in other studies [19, 20, 22].

Several major limitations of the study should be emphasized when interpreting the results. Responsive elevation of blood pressure was not uncommon after acute stroke especially in hemorrhagic stroke [23]. The blood pressure may rise as a result of increased intracranial pressure, pain, infection, psychological stress, urine retention, damage of brain regions that regulate the autonomic nerve tonic after stroke [24]. These responsive hypertensions were usually transient and the blood pressure, in most cases, would return to the baseline levels within the first week [25, 26]. To prevent misdiagnosed hypertension due to these conditions, blood pressure was monitored at least twice daily within 14 days of stroke onset. Because only 6 % of enrolled stroke patients were hemorrhagic in this study, we believe the total impact of inconsistency between pre-and post-stroke blood pressure in hemorrhagic stroke was minimal. In addition, hypotension caused by the index stroke (e.g., infarct in insula) may increase the likelihood of false negative in presuming a hypertension diagnosis immediately before the index stroke. But this condition is less common [27].

Cognitive impairments were common in the first few weeks after stroke [28]. Impaired consciousness may also present in stroke patients [29]. Both conditions may affect the evaluation of hypertension awareness at time of stroke onset. To minimize these confounding effects, we asked a relative or guardian who was familiar with the medical history of the patient to confirm the hypertension awareness before the index stroke. The questionnaire was conducted before blood pressure monitoring to exclude possible memory bias.

Finally, all patients in this study were enrolled from one center (Nanjing) in southeast China, where the socioeconomic development is noticeably more advanced, educational level higher and general healthcare status more advantageous than other parts of the country. Thus, the awareness of hypertension may be higher in this patient group than that in other populations.

## Conclusions

A substantial proportion (15.9 %) of Chinese patients with hypertension had not been aware of this covert risk until an overt stroke occurred. Hypertension unawareness was associated with lower educational levels and smoking, which address the importance of health education especially in these individuals.

## Abbreviations

SBP: systolic blood pressure
NSRP: Nanjing Stroke Registry Program
WHO: World Health Organization
DBP: diastolic blood pressure
DM: diabetes mellitus
BMI: body mass index
IQR: interquartile range
OR: odds ratio
CI: confidence interval
